# 4-(4-Amino-5-thioxo-4,5-dihydro-1*H*-1,2,4-triazol-3-yl)pyridinium chloride

**DOI:** 10.1107/S1600536808026123

**Published:** 2008-08-20

**Authors:** Xiao-Yan Ren, Fang-Fang Jian

**Affiliations:** a Microscale Science Institute, Weifang University, Weifang 261061, People’s Republic of China

## Abstract

The crystal structure of the title compound, C_7_H_8_N_5_S^+^·Cl^−^, is stabilized by inter­molecular N—H⋯Cl and N—H⋯S hydrogen-bond inter­actions.

## Related literature

For related literature, see: Jian *et al.* (2006 ▶); Shi *et al.* (1995 ▶); Xu *et al.* (2002 ▶).
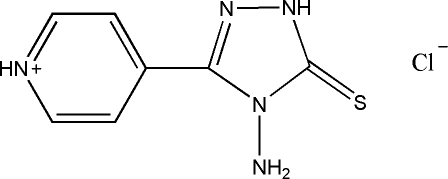


## Experimental

### 

#### Crystal data

C_7_H_8_N_5_S^+^·Cl^−^
*M* *_r_* = 229.69
Monoclinic, 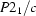

*a* = 7.6740 (15) Å
*b* = 13.374 (3) Å
*c* = 9.965 (2) Å
β = 104.70 (3)°
*V* = 989.3 (4) Å^3^
*Z* = 4
Mo *K*α radiation
μ = 0.56 mm^−1^
*T* = 293 (2) K
0.20 × 0.15 × 0.11 mm

#### Data collection

Enraf–Nonius CAD-4 diffractometer
Absorption correction: none
2238 measured reflections
2091 independent reflections
1712 reflections with *I* > 2σ(*I*)
*R*_int_ = 0.050
3 standard reflections every 100 reflections intensity decay: none

#### Refinement

*R*[*F* ^2^ > 2σ(*F* ^2^)] = 0.038
*wR*(*F* ^2^) = 0.111
*S* = 0.96
2091 reflections
135 parameters
H atoms treated by a mixture of independent and constrained refinement
Δρ_max_ = 0.35 e Å^−3^
Δρ_min_ = −0.29 e Å^−3^

### 

Data collection: *CAD-4 Software* (Enraf–Nonius, 1989 ▶); cell refinement: *CAD-4 Software*; data reduction: *NRCVAX* (Gabe *et al.*, 1989 ▶); program(s) used to solve structure: *SHELXS97* (Sheldrick, 2008 ▶); program(s) used to refine structure: *SHELXL97* (Sheldrick, 2008 ▶); molecular graphics: *SHELXTL/PC* (Sheldrick, 2008 ▶); software used to prepare material for publication: *WinGX* (Farrugia, 1999 ▶).

## Supplementary Material

Crystal structure: contains datablocks global, I. DOI: 10.1107/S1600536808026123/at2609sup1.cif

Structure factors: contains datablocks I. DOI: 10.1107/S1600536808026123/at2609Isup2.hkl

Additional supplementary materials:  crystallographic information; 3D view; checkCIF report

## Figures and Tables

**Table 1:** Hydrogen-bond geometry (Å, °)

*D*—H⋯*A*	*D*—H	H⋯*A*	*D*⋯*A*	*D*—H⋯*A*
N1—H1*A*⋯Cl1^i^	0.86	2.43	3.099 (2)	135
N3—H3*A*⋯Cl1^ii^	0.86	2.17	3.027 (2)	176
N5—H5*B*⋯S1^iii^	0.84 (3)	2.72 (3)	3.466 (3)	148 (3)

Symmetry codes: (i) 
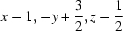
; (ii) 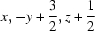; (iii) 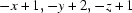.

**Fig. 1.**
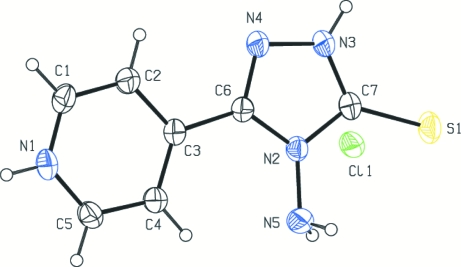
The molecular structure of the title compound with the atom-labeling scheme. Displacement ellipsoids are drawn at the 30% probability level.

## supplementary crystallographic information

### Comment

An important type of fungicides, triazole compounds are highly efficient and of low tocicity (Shi *et al.*,1995; Xu, *et al.*, 2002). The part of our research is to find triazole with higher cooperational activity, we synthesized the title compound (I) and report its crystal structure here.

In the crystal structure of compound (I) (Fig. 1), the dihedral angle formed by the triazole ring (N1/N3/N4/C6/C7) and the pyridine ring (N1/C1-C5) was 0.7 (4)°. The C═S bond length [1.676 (3) Å] is in agreement with that observed before (Jian, *et al.*, 2006). There are intermolecular N–H···Cl and N—H···S hydrogen-bond interactions to stabilize the crystal structure (Table 1).

### Experimental

The title compound (I) was prepared by the process as following: ethyl isonicotinate 1.51 g (0.01 mol) and hydrazine hydrate 0.32 g (0.01 mol) with ethanol at 377 K for 3 h, afford ivory-white compound A 1.32 g (yield 96%), then add 0.06 ml carbon disulfide and KOH 0.56 g (0.01 mol) with ethanol, stirred at room temperature for 5 h, afford yellow compound B 2.0 g (yield 85.6%). At last, add 0.32 g hydrazine hydrate to the compound B with water at 377 K for 12 h. Single crystals suitable for X-ray measurements were obtained by recrystallization from DMF-HCl (3:1) at 334 K.

### Refinement

The H atoms of the NH_2_ group were found from a difference Fourier map and refined freely. The other H atoms were positioned geometrically and allowed to ride on their parent atoms, with N—H and C—H distances of 0.86 and 0.93 Å, respectively, and with *U*_iso_(H) = 1.2*U*_eq_ of the parent atoms.

### Crystal data

**Table:**

C_7_H_8_N_5_S^+^·Cl^–^	*F*_000_ = 472
*M**_r_* = 229.69	*D*_x_ = 1.542 Mg m^−^^3^
Monoclinic, *P*2_1_/*c*	Mo *K*α radiation λ = 0.71073 Å
Hall symbol: -P 2ybc	Cell parameters from 25 reflections
*a* = 7.6740 (15) Å	θ = 4–14º
*b* = 13.374 (3) Å	µ = 0.56 mm^−^^1^
*c* = 9.965 (2) Å	*T* = 293 (2) K
β = 104.70 (3)º	Bar, yellow
*V* = 989.3 (4) Å^3^	0.20 × 0.15 × 0.11 mm
*Z* = 4	

### Data collection

**Table:**

Enraf–Nonius CAD-4 diffractometer	*R*_int_ = 0.050
Radiation source: fine-focus sealed tube	θ_max_ = 27.0º
Monochromator: graphite	θ_min_ = 2.6º
*T* = 293(2) K	*h* = 0→9
ω scans	*k* = 0→15
Absorption correction: none	*l* = −11→11
2238 measured reflections	3 standard reflections
2091 independent reflections	every 100 reflections
1712 reflections with *I* > 2σ(*I*)	intensity decay: none

### Refinement

**Table:**

Refinement on *F*^2^	Secondary atom site location: difference Fourier map
Least-squares matrix: full	Hydrogen site location: inferred from neighbouring sites
*R*[*F*^2^ > 2σ(*F*^2^)] = 0.038	H atoms treated by a mixture of independent and constrained refinement
*wR*(*F*^2^) = 0.111	*w* = 1/[σ^2^(*F*_o_^2^) + (0.0626*P*)^2^ + 0.6426*P*] where *P* = (*F*_o_^2^ + 2*F*_c_^2^)/3
*S* = 0.96	(Δ/σ)_max_ < 0.001
2091 reflections	Δρ_max_ = 0.35 e Å^−^^3^
135 parameters	Δρ_min_ = −0.29 e Å^−^^3^
Primary atom site location: structure-invariant direct methods	Extinction correction: none

### Special details

**Table:**

Geometry. All e.s.d.'s (except the e.s.d. in the dihedral angle between two l.s. planes) are estimated using the full covariance matrix. The cell e.s.d.'s are taken into account individually in the estimation of e.s.d.'s in distances, angles and torsion angles; correlations between e.s.d.'s in cell parameters are only used when they are defined by crystal symmetry. An approximate (isotropic) treatment of cell e.s.d.'s is used for estimating e.s.d.'s involving l.s. planes.
Refinement. Refinement of *F*^2^ against ALL reflections. The weighted *R*-factor *wR* and goodness of fit *S* are based on *F*^2^, conventional *R*-factors *R* are based on *F*, with *F* set to zero for negative *F*^2^. The threshold expression of *F*^2^ > σ(*F*^2^) is used only for calculating *R*-factors(gt) *etc*. and is not relevant to the choice of reflections for refinement. *R*-factors based on *F*^2^ are statistically about twice as large as those based on *F*, and *R*- factors based on ALL data will be even larger.

### Fractional atomic coordinates and isotropic or equivalent isotropic displacement parameters (Å^2^)

**Table:**

	*x*	*y*	*z*	*U*_iso_*/*U*_eq_	
Cl1	0.80625 (8)	0.86963 (4)	0.12228 (5)	0.04585 (18)	
S1	0.75196 (9)	0.94026 (5)	0.52007 (7)	0.0565 (2)	
N1	0.0222 (2)	0.78167 (16)	−0.16338 (19)	0.0469 (5)	
H1A	−0.0577	0.7735	−0.2405	0.056*	
N2	0.4905 (2)	0.89786 (14)	0.28259 (18)	0.0376 (4)	
N3	0.5917 (2)	0.76955 (15)	0.40203 (19)	0.0452 (5)	
H3A	0.6505	0.7315	0.4676	0.054*	
N4	0.4694 (3)	0.73433 (15)	0.28792 (19)	0.0439 (4)	
N5	0.4688 (4)	0.99550 (16)	0.2289 (3)	0.0540 (6)	
C1	0.0851 (3)	0.7017 (2)	−0.0868 (2)	0.0494 (6)	
H1B	0.0430	0.6383	−0.1173	0.059*	
C2	0.2114 (3)	0.71260 (18)	0.0365 (2)	0.0459 (5)	
H2A	0.2551	0.6568	0.0903	0.055*	
C3	0.2744 (3)	0.80777 (17)	0.0811 (2)	0.0362 (5)	
C4	0.2062 (3)	0.88900 (18)	−0.0017 (2)	0.0441 (5)	
H4B	0.2462	0.9533	0.0254	0.053*	
C5	0.0786 (3)	0.87364 (19)	−0.1248 (2)	0.0491 (6)	
H5C	0.0318	0.9278	−0.1810	0.059*	
C6	0.4102 (3)	0.81438 (16)	0.2152 (2)	0.0367 (5)	
C7	0.6114 (3)	0.86845 (18)	0.4025 (2)	0.0402 (5)	
H5A	0.575 (5)	1.011 (3)	0.211 (4)	0.092 (12)*	
H5B	0.443 (4)	1.032 (2)	0.290 (3)	0.065 (9)*	

### Atomic displacement parameters (Å^2^)

**Table:**

	*U*^11^	*U*^22^	*U*^33^	*U*^12^	*U*^13^	*U*^23^
Cl1	0.0495 (3)	0.0475 (3)	0.0356 (3)	0.0015 (2)	0.0015 (2)	0.0021 (2)
S1	0.0499 (4)	0.0593 (4)	0.0497 (4)	−0.0019 (3)	−0.0071 (3)	−0.0162 (3)
N1	0.0392 (10)	0.0627 (13)	0.0340 (10)	−0.0050 (9)	0.0007 (7)	−0.0061 (9)
N2	0.0357 (9)	0.0395 (9)	0.0353 (9)	−0.0011 (7)	0.0044 (7)	−0.0037 (7)
N3	0.0436 (10)	0.0493 (11)	0.0356 (9)	−0.0030 (9)	−0.0030 (8)	0.0022 (8)
N4	0.0440 (10)	0.0465 (11)	0.0354 (9)	−0.0058 (8)	−0.0004 (8)	−0.0004 (8)
N5	0.0633 (15)	0.0395 (12)	0.0503 (13)	0.0003 (10)	−0.0024 (11)	−0.0020 (10)
C1	0.0500 (13)	0.0506 (14)	0.0445 (12)	−0.0123 (11)	0.0063 (10)	−0.0096 (11)
C2	0.0486 (12)	0.0445 (13)	0.0402 (12)	−0.0047 (10)	0.0035 (9)	0.0002 (10)
C3	0.0324 (10)	0.0451 (12)	0.0317 (10)	−0.0027 (9)	0.0090 (8)	−0.0032 (9)
C4	0.0464 (12)	0.0421 (12)	0.0397 (11)	−0.0016 (10)	0.0034 (9)	−0.0017 (9)
C5	0.0498 (13)	0.0509 (14)	0.0403 (12)	0.0037 (11)	0.0000 (10)	0.0018 (10)
C6	0.0340 (10)	0.0409 (11)	0.0350 (10)	−0.0026 (8)	0.0081 (8)	−0.0009 (8)
C7	0.0348 (10)	0.0491 (13)	0.0347 (11)	−0.0002 (9)	0.0055 (8)	−0.0043 (9)

### Geometric parameters (Å, °)

**Table:**

S1—C7	1.679 (2)	N5—H5A	0.90 (4)
N1—C5	1.328 (3)	N5—H5B	0.84 (3)
N1—C1	1.331 (3)	C1—C2	1.366 (3)
N1—H1A	0.8600	C1—H1B	0.9300
N2—C6	1.366 (3)	C2—C3	1.394 (3)
N2—C7	1.371 (3)	C2—H2A	0.9300
N2—N5	1.405 (3)	C3—C4	1.385 (3)
N3—C7	1.331 (3)	C3—C6	1.474 (3)
N3—N4	1.362 (3)	C4—C5	1.376 (3)
N3—H3A	0.8600	C4—H4B	0.9300
N4—C6	1.308 (3)	C5—H5C	0.9300
			
C5—N1—C1	122.28 (19)	C1—C2—H2A	120.2
C5—N1—H1A	118.9	C3—C2—H2A	120.2
C1—N1—H1A	118.9	C4—C3—C2	118.6 (2)
C6—N2—C7	108.35 (18)	C4—C3—C6	124.5 (2)
C6—N2—N5	125.26 (18)	C2—C3—C6	116.9 (2)
C7—N2—N5	125.94 (19)	C5—C4—C3	119.3 (2)
C7—N3—N4	113.60 (18)	C5—C4—H4B	120.3
C7—N3—H3A	123.2	C3—C4—H4B	120.3
N4—N3—H3A	123.2	N1—C5—C4	120.1 (2)
C6—N4—N3	104.37 (18)	N1—C5—H5C	119.9
N2—N5—H5A	105 (2)	C4—C5—H5C	119.9
N2—N5—H5B	107 (2)	N4—C6—N2	110.29 (18)
H5A—N5—H5B	114 (3)	N4—C6—C3	121.29 (19)
N1—C1—C2	120.1 (2)	N2—C6—C3	128.43 (19)
N1—C1—H1B	120.0	N3—C7—N2	103.35 (18)
C2—C1—H1B	120.0	N3—C7—S1	128.55 (17)
C1—C2—C3	119.6 (2)	N2—C7—S1	128.10 (18)

### Hydrogen-bond geometry (Å, °)

**Table:**

*D*—H···*A*	*D*—H	H···*A*	*D*···*A*	*D*—H···*A*
N1—H1A···Cl1^i^	0.86	2.43	3.099 (2)	135
N3—H3A···Cl1^ii^	0.86	2.17	3.027 (2)	176
N5—H5B···S1^iii^	0.84 (3)	2.72 (3)	3.466 (3)	148 (3)

Symmetry codes: (i) *x*−1, −*y*+3/2, *z*−1/2; (ii) *x*, −*y*+3/2, *z*+1/2; (iii) −*x*+1, −*y*+2, −*z*+1.

## References

[bb1] Enraf–Nonius (1989). *CAD-4 Software* Enraf–Nonius, Delft, The Netherlands.

[bb2] Farrugia, L. J. (1999). *J. Appl. Cryst.* **32**, 837–838.

[bb3] Gabe, E. J., Le Page, Y., Charland, J.-P., Lee, F. L. & White, P. S. (1989). *J. Appl. Cryst.* **22**, 384–387.

[bb4] Jian, F.-F., Yu, H.-Q., Qiao, Y.-B. & Liang, T.-L. (2006). *Acta Cryst.* E**62**, o3416–o3417.

[bb5] Sheldrick, G. M. (2008). *Acta Cryst.* A**64**, 112–122. 10.1107/S0108767307043930 18156677

[bb6] Shi, Y. N., Lu, Y. C. & Fang, J. X. (1995). *Chem. J. Chin. Univ.* **16**, 1710–1713.

[bb7] Xu, L. Z., Zhang, S. S., Li, H. J. & Jiao, K. (2002). *Chem. Res. Chin. Univ.* **18**, 284–286.

